# Magnetization–structure–composition phase diagram mapping in Co-Fe-Ni alloys using diffusion multiples and scanning Hall probe microscopy

**DOI:** 10.1038/s41598-022-05121-1

**Published:** 2022-02-04

**Authors:** Girfan Shamsutdinov, Peng Zhao, Sreenivas Bhattiprolu, Ji-Cheng Zhao, Boris Nadgorny

**Affiliations:** 1 Department of Physics and Astronomy, Wayne State University, 666 W. Hancock Rd, Detroit, MI 48201 USA; 2 Department of Materials Science and Engineering, The Ohio State University, 2041 N College Rd, Columbus, OH 43210 USA; 3 Oxford Instruments America, Inc., Concord, MA 01742 USA; 4 Carl Zeiss X-Ray Microscopy, Inc., 5300 Central Parkway, Dublin, CA USA; 5 Department of Materials Science and Engineering, University of Maryland College Park, 4418 Stadium Drive, College Park, MD 20742-2115 USA

**Keywords:** Materials science, Physics

## Abstract

Transition metal alloys are essential for magnetic recording, memory, and new materials-by-design applications. Saturation magnetization in these alloys have previously been measured by conventional techniques, for a limited number of samples with discrete compositions, a laborious and time-consuming effort. Here, we propose a method to construct complete saturation magnetization diagrams for Co–Fe–Ni alloys using scanning Hall probe microscopy (SHPM). A composition gradient was created by the diffusion multiple technique, generating a full combinatorial materials library with an identical thermal history. The composition and crystallographic phases of the alloys were identified by integrated energy dispersive X-ray spectroscopy and electron backscatter diffraction. “Pixel-by-pixel” perpendicular components of the magnetic field were converted into maps of saturation magnetization using the inversion matrix technique. The saturation magnetization dependence for the binary alloys was consistent with the Slater-Pauling behavior. By using a significantly denser data point distribution than previously available, the maximum of the Slater-Pauling curve for the Co–Fe alloys was identified at ~ 32 at% of Co. By mapping the entire ternary diagram of Co–Fe–Ni alloys recorded in a single experiment, we have demonstrated that SHPM—in concert with the combinatorial approach—is a powerful high-throughput characterization tool, providing an effective metrology platform to advance the search for new magnetic materials.

## Introduction

**Figure 1:**
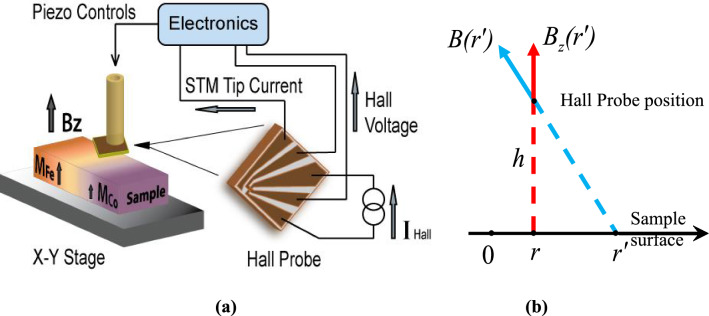
(**a**) Schematics of the measurement setup: An HP is scanned in the lift-off mode across the surface of a variable composition alloy starting from pure Fe (here, an Fe–Co binary alloy is shown) with the external magnetic fields applied in the ***z***-direction to saturate the magnetization of the alloy; (**b**) A map of the* z*-component of the magnetic field is generated by the SHPM scanned at height *h* above the sample surface and is converted to magnetization by using a simple dipole model.

Traditionally, materials property mapping as a function of composition and structure was accomplished through laborious and time-consuming synthesis and analysis of samples with discrete compositions prepared one at a time. One of the drawbacks of this approach is the assumption—which may or may not be justified—that the set of conditions used in different sample fabrications is identical; it is also inherently limited in its compositional resolution, which may adversely affect its effectiveness as a tool for new material discovery. An alternative *combinatorial materials science approach*, on the other hand, has shown promise to accelerate material discovery^1–12^. The main focus of this approach to date is to combine parallel synthesis with rapid characterization techniques to map out physical or chemical properties as functions of composition for a given system. Mapping out more complex relationships between lattice structure and physical properties for different compositions in a multicomponent system in one combinatorial experiment remains a challenge. Addressing this challenge, herein, we present a detailed map of the crystal structure and physical properties of Co–Fe, Fe–Ni, Co–Ni binary, and Co–Fe–Ni ternary alloys with continuous composition spread in a single bulk sample. Co–Fe–Ni binary and ternary alloys have been the subject of intense attention for many years due to their importance to the physics of magnetism as well as applications in magnetic recording^13^, as well as their potential for the fabrication of hard rare earth-free magnets^14^. Theoretically, the saturation magnetization in transition metal ferromagnets Co, Fe, Ni and their alloys is fairly well described by the well-known Slater-Pauling curve^15,16^, which justifies the continuous dependence of the saturation magnetization on the number of 3d- and 4s-electrons per atom. Experimentally, however, only a limited set of data is available in the literature, and magnetic phase diagrams of bulk alloys have not been systematically investigated. The issue is compounded by the fact that the crystallographic structure of these alloys, which can significantly affect their magnetic properties^15,16^, is strongly dependent on the thermal history and the fabrication technique used for sample preparation. To avoid these problems, we utilized the well-established diffusion multiples fabrication technique to prepare fully characterized Co–Fe–Ni samples, each of which contains the entire single-phase composition spread of binary and ternary alloys. While magnetization studies of similar systems, such as Fe–Ni and Co–Fe–Ni diffusion films, were performed with the SHPM instrument by Yoo et al.^17,18^, the low applied magnetic fields used in these experiments (on the order of 150 Oe) did not allow measurements of saturation magnetization. The Co–Fe–Ni alloys in this study were fabricated by annealing three metal blocks placed in intimate contact at high temperatures to allow thermal interdiffusion to create a solid-state solution with a composition spread over the binary and ternary diffusion regions. We used a scanning Hall probe microscope (SHPM) in a lift-off mode under ambient conditions to investigate and map the Co–Fe–Ni saturation magnetization by detecting magnetic field distributions near the surface of the sample with variable composition, with resolution limited only by the sample and the Hall Probe geometry (see Fig. 1). By applying the dipole approximation to these experimental data, we determined the saturation magnetizations of the alloys at any given point/composition. The values of the saturation magnetization were found to be in good agreement with the known values for elemental Fe, Co and Ni metals and their alloys, demonstrating the robustness of the technique. As the saturation magnetization of Co–Fe–Ni alloys is known to be dependent not only on the chemical composition but also on the crystallographic structure, we used energy dispersive X-ray spectroscopy (EDS) and electron backscatter diffraction (EBSD) to augment the magnetic data with the composition variations and the crystal phase structure of the corresponding area.

## Sensing principle and modeling

### Scanning Hall probe microscopy

The magnetic field in the vicinity of a sample surface is uniquely determined by the sample magnetization. Several techniques, such as Bitter decoration^19^, the Faraday rotation effect^20^, Lorenz microscopy and electron holography^21^, magnetic force microscopy (MFM)^22^ and Hall probe sensing^23^, have been employed to detect surface magnetic fields. While each of these techniques has distinctive advantages, SHPM combines many desirable features: it is noninvasive, has an excellent, potentially submicron spatial resolution and allows quantitative measurements of large magnetic fields with high sensitivity (no saturation). The last feature is particularly important for saturation magnetization measurements, which require applications of considerable magnetic fields. The schematics of the experimental setup for the saturation magnetization mapping of variable composition magnetic materials is shown in Fig. 1a. The Hall probe is set up in close proximity to the sample surface, and then a scan across a binary or over a ternary diffusion region is performed. During the line scan, the sample holder (stage) is moved step by step along a straight line across the diffusion region where the composition of the alloy changes, for example, from pure Co on one side to pure Fe on the other side of the sample (as shown in Fig. 1a). The Hall probe is sensitive to the *z*-component of the stray magnetic field at the surface of the sample and generates a Hall voltage, *V*_*Hall*_ , when a Hall current, *I*_*Hall *_ is passed through the probe. The sample is magnetized to saturation along the *z*-direction. Thus, the Hall probe measures the resultant *z* component of the magnetic field due to magnetization of the sample and the applied field, which can be easily subtracted. The saturation magnetization values can be extracted from the magnetic field distribution along the scan line. The SHPM Hall sensor was scanned over a diffusion couple/multiple sample in the liftoff mode (at constant height), and the results were recorded as a function of the Hall probe position.

The source of the perpendicular (*z*-component) of the magnetic field measured by the SHPM can be understood by considering contributions from regions of different compositions within a simple dipole approximation. If we assume that the magnetization of the sample can be represented as a surface magnetic pole charge density *M*_*S*_*(r)*, then the perpendicular component of the magnetic field at distance *h* above the surface is given by $B_{z} (r) = \int\limits_{{r^{\prime}}} {\frac{h}{{\left[ {\left( {r - r^{\prime}} \right)^{2} + h^{2} } \right]^{3/2} }}M_{S} (r^{\prime})} dr^{\prime}$, see Fig. 1b. Since the outcome of our measurements is a discrete set of *B*_*z*_ values *B*_z_(*r*_i_), where *r*_*i*_ is the position of the measured point *i* along the scan trajectory and *Δr* is the distance between two adjacent measurement points, we can replace the integral with the sum ${B}_{z}\left({r}_{i}\right)={\sum }_{ij}\frac{h}{{{[\left({r}_{i}-{r}_{j}\right)}^{2}+{h}^{2}]}^{3/2}}{M}_{s}\left({r}_{j}\right)\Delta r$. To find the magnetization at each measurement point along the scan line, we need to solve the inverse problem, i.e., to solve the matrix equation *B*_*zi*_ = *D*_*ij*_*M*_*sj*_*,* where $D_{ij} = \frac{h}{{\left[ {\left( {r_{i} - r_{j} } \right)^{2} + h^{2} } \right]^{3/2} }}$ Δr. Thus, the value of the magnetic pole charge density at point *i* along the scan line is given by *M*_*si*_ = *(D*^*-1*^*)*_*ij*_*B*_*zj*_, where the geometric factors *(D*^*-1*^*)*_*ij*_ are the elements of the inverse matrix *D*^*-1*^*.* This proportionality coefficient does not change as long as the scan is performed far from the sample edges and the probe-surface distance remains the same. To calibrate the probe, we position it over the area of the sample with known magnetization, which then allows us to determine the magnetization values for the rest of the sample. For our diffusion multiple Co–Fe–Ni samples, we set the probe over a point within a pure Fe region in the beginning of the scan and use that point as a reference for magnetization calculations (see Fig. 1a). The details of the Hall Probe calibration are described in the Supplementary section (Fig. S1).

### Diffusion multiples and the composition–structure–property relationship

A *diffusion multiple* is an assembly of three or more different metal blocks that are brought in close interfacial contact and exposed to a high temperature to allow thermal interdiffusion^24–28^ The interdiffusion among the Co–Ni–Fe blocks (Fig. 7) produces complete libraries of the intermetallic compounds and solid-solution phases in this system. In combination with advanced microanalytical techniques such as EDS and EBSD, composition libraries make diffusion multiples a powerful approach for rapid mapping of phase diagrams and materials properties for multicomponent alloy systems. To determine the composition–structure–property relationship for Co–Fe–Ni alloys with variable compositions, we investigate the composition and structure variation across the binary and ternary diffusion regions and relate it to the crystal structure and the magnetic properties of the samples.

## Results

### Elemental composition analysis

**Figure 2:**
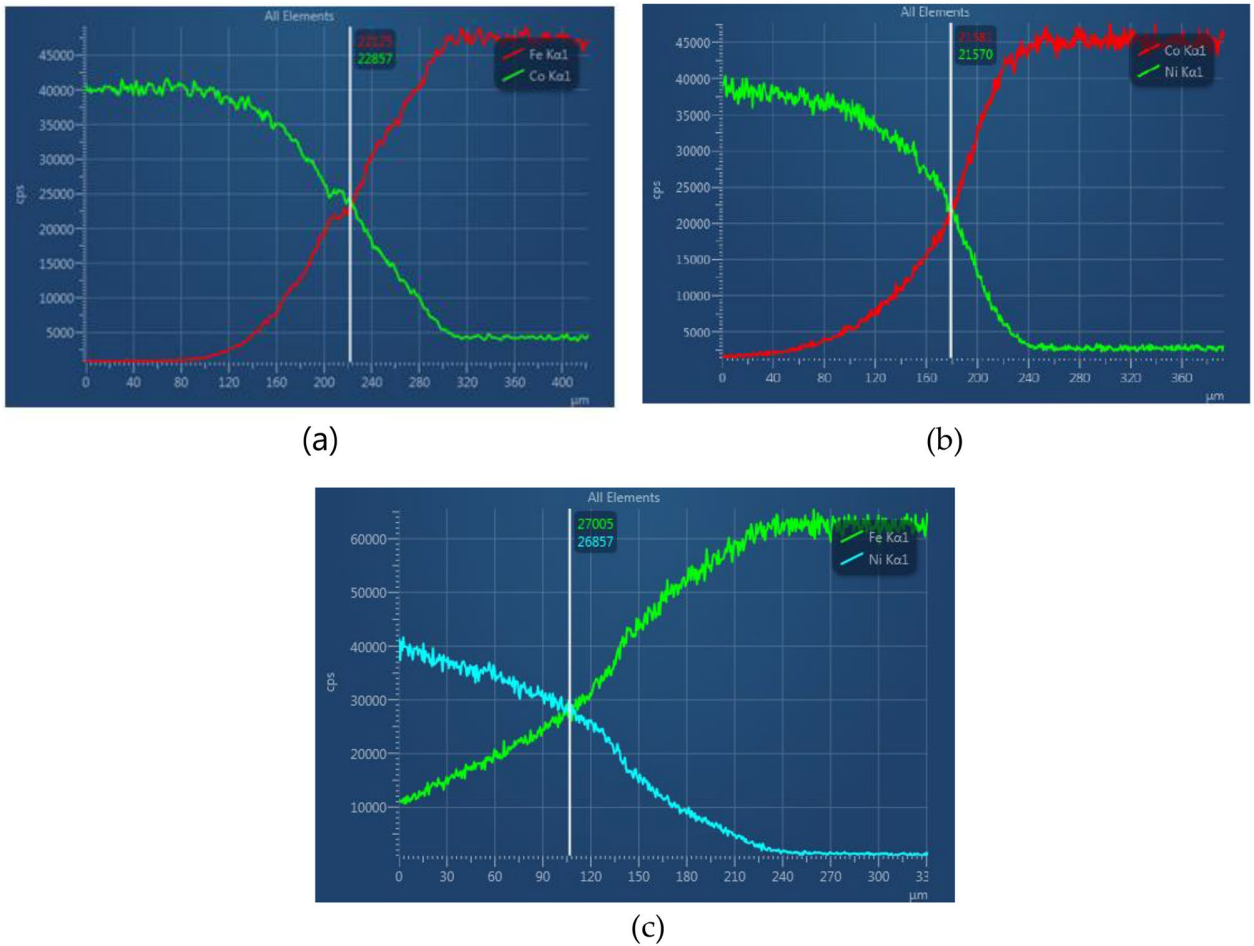
EDS line scans, K_α_ *X*-ray counts per second (cps) vs. distance (µm), indicating composition variations in different diffusion couples: (**a**) composition variation in a Co (green)—Fe (red) diffusion couple; (**b**) composition variation in Co (red)—Ni (green) couple; and (**c**)) composition variation in Fe (green)—Ni (blue) couple. Note that the composition variation is inferred from the Ka X-ray intensity for a given element. A true weight percentage calculation for each element requires a comparison of X-ray intensities against known elemental standards followed by corrections for absorption and fluorescence by the sample matrix.

To perform elemental and structural analysis, Oxford Instruments X-Max EDS detector with a large area analytical Silicon Drift EDS Detector (SDD) and Oxford Instruments Nordlys EBSD detector were used. As the first step of characterizing the diffusion of multiple interfaces, we performed a series of line scans across the three binary regions (Co–Fe, Co–Ni, and Ni–Fe) using scanning electron microscopy (SEM). The electron beam of the SEM equipped with the X-ray EDS detector was scanned across the respective diffusion binary interfaces with a step size of approximately 1.3 µm to ensure the necessary spatial resolution. The linear scan graphs for binary diffusion interfaces represent the number of characteristic K_α_ X-ray counts vs. distance across the respective interface (Fig. 2). For our Co–Fe–Ni samples, the diffusion lengths are approximately 240 μm for Co–Fe and Co–Ni and 340 μm for Fe–Ni junctions, with an estimated uncertainty of less than 5%. An example of line scans is shown in Fig. 2.

### Integrated EBSD/EDS measurements for sample crystal structure determination

**Figure 3:**
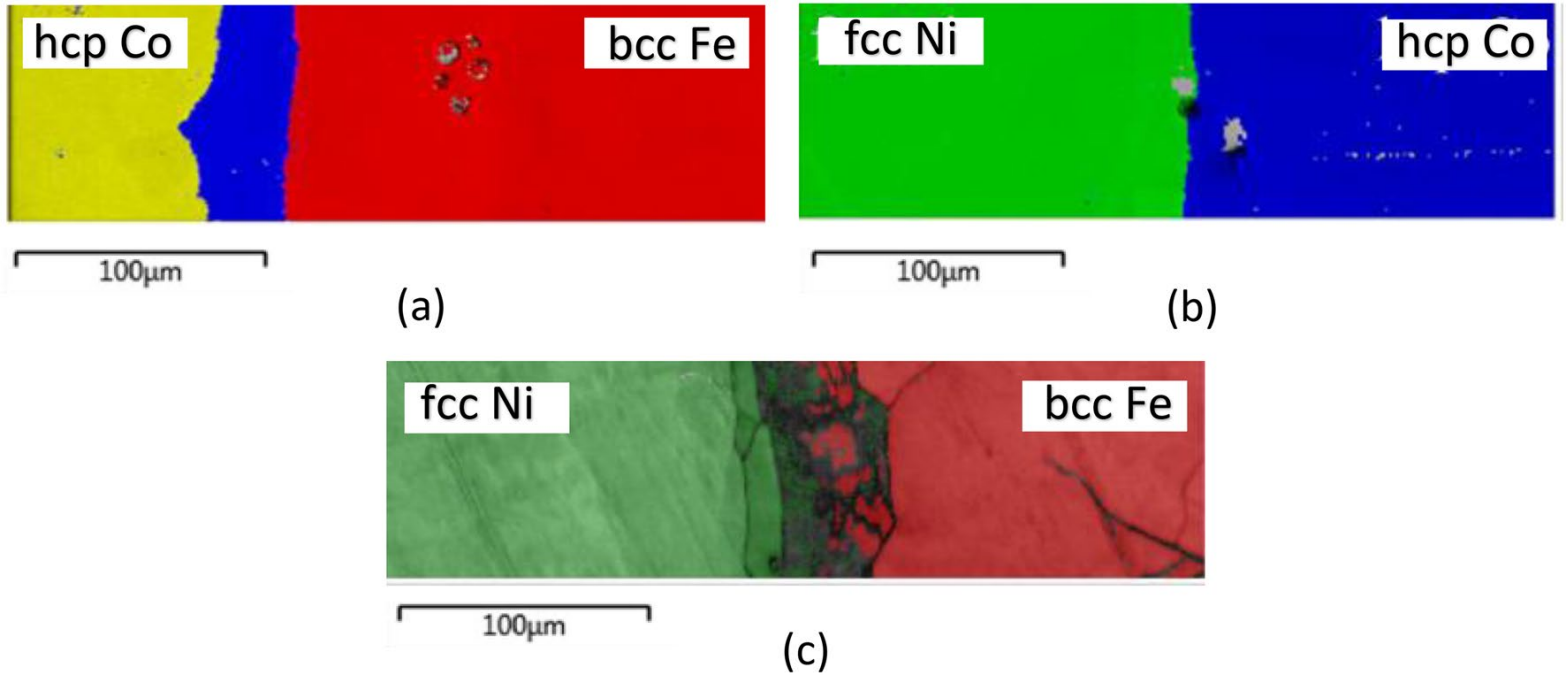
Crystal phase distribution in binary regions produced by integrated EBSD/EDS data analysis: (**a**) Co–Fe couple revealing binary regions with hcp phase (yellow), fcc (blue), and bcc (red); Ni–Co binary region revealing fcc (green) and hcp (blue) phases; (**c**) Ni–Fe binary region with fcc (green) and bcc (red) phases.

The physical properties of many materials (including magnetic properties) often depend not only on the composition but also on the crystal structure (phase). Modern EBSD systems are highly accurate and offer orientation angular resolutions less than 0.5 degrees. However, EBSD alone is insufficient for identification of a metallurgical phase, as it requires information about the chemical composition of the sample. Similarly, EDS alone cannot be used, as phase identification requires crystallographic information. Therefore, to accurately identify all phases in Co–Fe–Ni multiples, integrated EBSD/EDS analysis was performed. The basic idea behind the integrated approach is to simultaneously collect EBSD and EDS data and then to process the data offline using recorded EDS data as a filter to assist in the standard phase differentiation process by indexing individual EBSD patterns. When simultaneous EBSD/EDS collection can be performed, the capabilities of both techniques can be significantly enhanced, and the accuracy of crystal phase and composition identification can be markedly improved. This is particularly relevant for the Fe–Co system, where it is difficult to distinguish between the bcc, fcc, and hcp phases of Co and Fe, as the sample composition gradually changes and the phase determination by EBSD alone is not reliable. Integrated EBSD/EDS mapping of Co–Fe–Ni couples and multiples was performed over the ternary and binary areas of the sample. The crystal phase maps for the Co–Fe, Ni–Fe, and Ni–Co couples are shown in Fig. 3.

**Figure 4:**
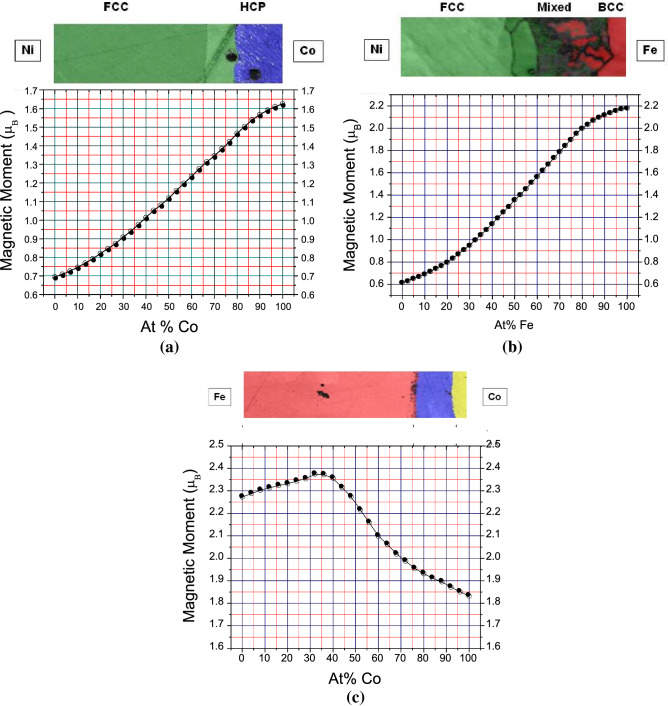
(**a**) Saturation magnetization across the Ni-Co diffusion zones (bottom) combined with the structural phase diagram of equilibrated Co–Ni binary alloy obtained by integrated EBSD/EDS analysis (top) revealing fcc (green) and hcp (blue) phases. (**b**) Saturation magnetization across the Fe–Ni diffusion zones (bottom) combined with the structural phase diagram of equilibrated Fe–Ni binary alloy obtained by integrated EBSD/EDS analysis (top) revealing fcc (green), bcc (red), and mixed fcc/bcc (dark gray) phases. (**c**) Saturation magnetization across the Co–Fe binary diffusion zone (bottom) combined with the structural phase diagram obtained by integrated EBSD/EDS analysis (top).

The finalized EBSD/EDS maps corresponding to the diffusion zones for Co–Fe, Fe–Ni, and Co–Ni are presented in concert with the magnetization data in Fig. 4. For the Co–Fe diffusion zone, the Fe bcc phase extends from the iron-rich side through the Fe bcc ordered alloy region up to approximately 25 at% Fe. The fcc Fe and Co phases are observed from 25 at% to 5 at% iron. The cobalt-rich region exhibits the hcp structure that forms at temperatures below 420 °C and can extend up to 6 at% Co^29^. The Ni–Fe binary system data show the (γFe, Ni) solid solution based on fcc Fe and Ni down to approximately 45 at% Ni on the nickel-rich side. The (αFe) solid solution based on the low-temperature bcc Fe exists up to ~ 6 at% Ni in the iron-rich region. Between 6 at% and 45 at% Ni, the (αFe) and (γFe, Ni) phases coexist in a mixed fcc-bcc solid solution. The latter region appears dark gray in the Fe–Ni EBSD map. Proper indexing of this region was demanding due to possible martensitic transformations in Ni–Fe alloys^30^, chemical inhomogeneity, and material deformation near the interface^31^. The Co–Ni binary region consists of fcc Co and Ni-based (γCo, Ni) and hcp (εCo) phases, with a phase boundary at ~ 15 at% Ni.

### Saturation magnetization measurements with scanning Hall probe microscopy (SHPM)

Magnetic measurements were performed at 300 K using the Low Temperature Scanning Hall Probe Microscope/Scanning Tunneling Microscope (LT-SHPM/STM) from *NanoMagnetics Instruments* in the Physical Property Measurement System (PPMS Model 6000) from *Quantum Design*. The applied magnetic field was approximately 5 T (49 kOe), sufficient to saturate all of the Co–Fe–Ni alloys. The Hall probe, sensitive to the component of the magnetic field perpendicular to the sample surface, *B*_*z*_*(r),* was scanned across the sample in small discrete steps (~ 5 μm). Both line and surface scans were performed. To extract the saturation magnetization values, we adopted—with some modifications—the so-called inversion technique developed for scanning SQUID microscope measurements of discrete magnetic libraries^32^, in which every sample from the library was assumed to be uniform. In the case of Co–Ni–Fe diffusion samples, the composition and hence the saturation magnetization changed continuously. However, as this change was slow, we assumed that within a single Hall probe step, the magnetization was uniform. To test the validity of our model, we took an arbitrary distribution of magnetization values and calculated the corresponding magnetic fields at every point of this “scan” line. We then took these simulated measurement results and converted them back to magnetization values, obtaining (with proper boundary conditions) almost exact reproduction of the initial model magnetization.

The magnetization value is then directly proportional to the value of the magnetic pole charge density (as described above), the proportionality coefficient does not change as long as the scan is performed far from the sample edges (no significant change in shape demagnetization coefficient), and the probe-surface distance remains the same. Therefore, after we calibrate the Hall probe over a particular area of the sample with known magnetization, we can find the values of magnetization for the rest of the sample. For our Co–Fe–Ni diffusion sample, for example, we can set the probe at a certain distance over a point in the iron-only region in the beginning of the scan and use that point as a reference for the magnetization calculations. Similarly, the saturation magnetization data obtained from the areas of pure Co, Fe, and Ni were used for SHPM calibration. The Hall probe calibration is described in Fig. S1 (Supplemental Materials). The experimental saturation magnetization results obtained from the Co–Fe–Ni diffusion samples agree (within 5%) with the accepted values for pure Co, Fe, and Ni, as presented in Table SI. The saturation magnetization results for Ni–Fe, Co–Ni, and Co–Fe binary systems augmented by crystal phase structure measurements are presented in Fig. 4a–c.

### Complete magnetic phase diagram of Ni–Fe–Co ternaries

**Figure 5:**
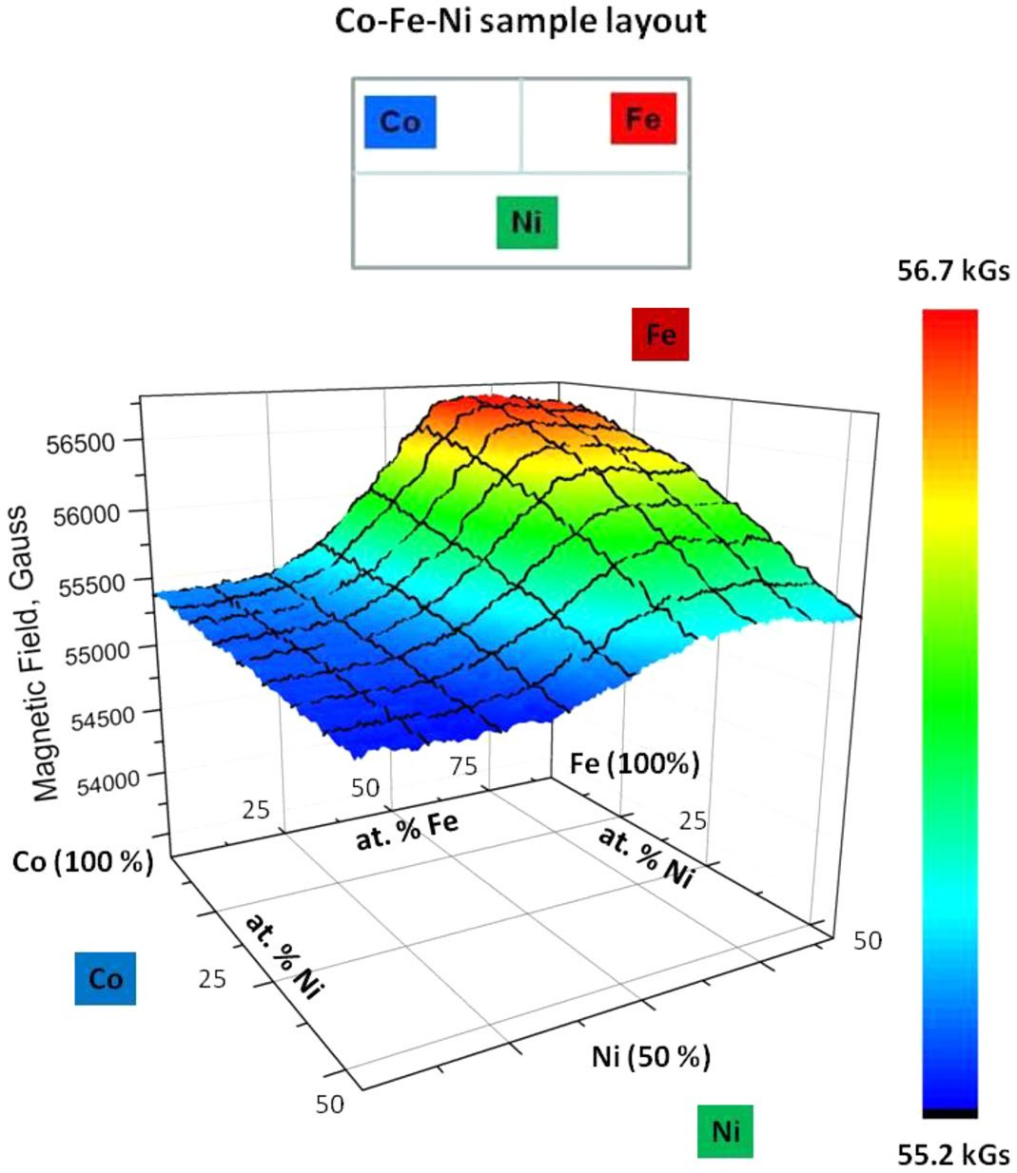
The sum of the applied magnetic field and the field generated by the magnetic alloys plotted over the Co–Fe–Ni ternary diffusion zone; the sample layout is shown on top. The area shown is approximately 150 μm along the X-axis by 300 μm along the Y-axis (from the Co side to the Fe side).

**Figure 6:**
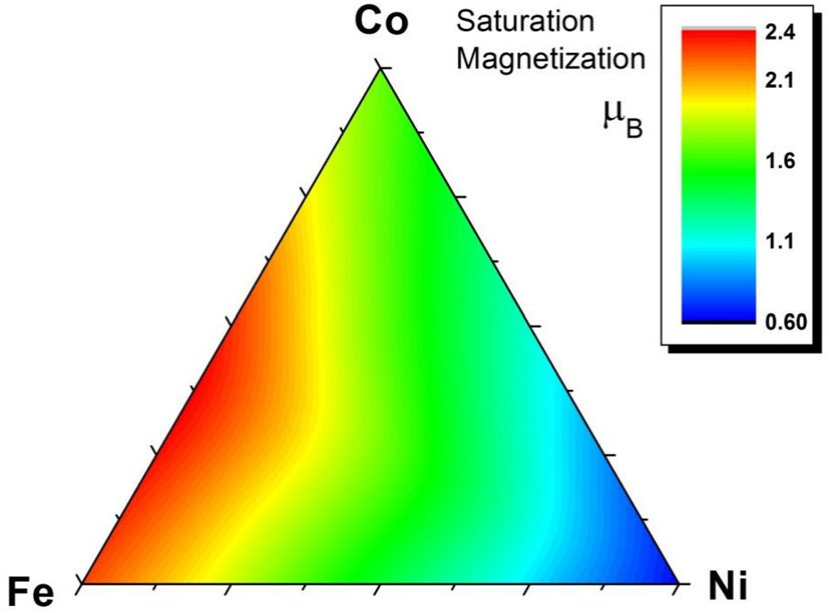
Saturation magnetization diagram mapped over the Co–Fe–Ni ternary diffusion zone.

Finally, after ensuring that all our data for the respective binary systems are consistent, we used the compendium of data obtained from line and surface scans to construct a comprehensive magnetic phase diagram for the Co–Fe–Ni ternary alloys fabricated by the diffusion multiple technique, as shown in Figs. 5, 6. Importantly, all the data for the Co–Fe–Ni magnetization measurements were performed as a single scan over the ternary alloy to ensure that the SHPM Hall probe surface distance, measured before and after the scan, did not change. The data are presented as a 2D magnetic field plot as a function of composition (Fig. 5), as well as a conventional ternary phase diagram of saturated magnetization in terms of Bohr magnetons, *µ*_*B*_ (Fig. 6), which summarizes the results of our magnetic saturation measurements of the entire Co–Fe–Ni system with identical thermal modification history.

## Discussion

Our results for all three binary alloys from EBSD/EDS maps are in good agreement with the existing data from equilibrated alloy diagrams in the 400 °C isothermal sections (see Ref.^29^). While the Co–Fe–Ni diffusion sample was annealed at 1000 °C, it was subsequently quenched in water, in an effort to retain the solid solution phases formed at 1000 °C to ambient temperature. However, phase transformation did occur in some composition regions to form the ambient temperature phases. In contrast to the majority of studies of bulk alloys, in which each composition point is processed under different thermal conditions, the current diffusion multiple sample represents a unique phase diagram of the Co–Fe–Ni alloy system with a single universal thermal history applied to all the compositions. The structural phases of Ni-Co, Ni–Fe, and Fe–Co binaries are in good qualitative agreement with the reports in the literature^33^. Quantitatively, we note that for bulk Ni–Fe alloys the mixed fcc-bcc phase is typically reported between 20 and 30 at% of Ni, where is in our diffusion multiples, it is broader (from 6 to 45 at% of Ni) and less uniform, as seen in Fig. 4b. Two main factors are likely to be contributing to poor EBSD pattern quality: (i) small grain sizes from the mixed phase is too small (ii) the presence of martensitic phase with high dislocation density and residual stress.

The magnetic properties of the bulk Ni–Co–Fe binary alloys as well as elemental Fe, Ni, and Co, are well-known, allowing us to calibrate SHPM and make sure the results are accurate. The saturation magnetization values are expected to correlate with the Slater-Pauling curves. For the Ni-Co system the measured saturation magnetization is essentially linear, in good agreement with the literature. On the other hand, in the Ni–Fe system the curve linearity (beyond the Fe bcc phase, where *M*_*s*_ is almost constant) is normally affected by phase transitions from the bcc to the mixed bcc–fcc to the fcc phases. However, in our case the mixed phase region is considerably broader, making the central part of the curve almost linear as well. The most interesting case is in the Fe–Co system, where the maximum value of *M*_*s*_ is indicative of the maximum electronic concentration in the range between Fe and Co. Our results for the Co–Fe system (Fig. 4c) are in good agreement with earlier measurements of bulk alloys with discrete compositions^29,34^, covering two separate regions: up to ~ 70% Co (bcc) and ~ 70–100% Co (fcc). Some discrepancy is expected, as the saturation magnetization is somewhat sensitive to the degree of disorder, which in turn depends on the cooling/quenching routine following sample annealing. From this perspective, our results should be compared to the results obtained for similarly quenched samples^34^. Our measurements suggest that this maximum corresponds to ~ 32 at% of Co, somewhat higher than the results of Ref. ^34^ (28%) but lower than the first measurement of this peak described in Ref.^35^ (35%) for slowly cooled samples, which were presumably more ordered^34^. We argue that some of the advantages of the SHPM-enabled technique, allow one to perform these measurements more accurately, which is important in this case, as the maximum in the experimental curve is not very pronounced. First, while the samples studied in Ref. ^34^ and other earlier works were prepared and quenched individually, all of our variable composition data points were obtained from the same sample and thus had identical thermal history. Second, while samples studied in Ref.^34^ were separated by 10 at% or more in terms of compositions, our data points were much denser, nominally separated by 1.5–2 at% (based on the 5 µm steps between consecutive measurement points and depending on the length of the diffusion region). While the actual compositional resolution of SHPM may be affected by the contributions of the adjacent areas of the sample (see the Supplemental section), it is still likely to be several times higher. Moreover, the closer inspection of the results of Ref.^34^, indicates that no measurements were performed between ~ 28 at% and ~ 40 at% of Co. The author interpolated the results by using a symmetric fit of the available data points, with the maximum at 28 at% of Co. However, there was no justification for this assumption and, in fact, our higher density measurements indicate that the curve is not symmetric near 28 at% of Co. Our data on FeCo (as well as other bulk alloys) are also in quantitative agreement with the measurements of ultrathin 3d transition-metal binary alloys^36^. Obtaining precise results with SHPM for the composition of the saturation magnetization maximum is important for a comparison with the Slater-Pauling curve^36^, average atomic volume calculations in Fe–Co and Fe–Ni alloys^37^, as well as first principle band structure calculations in transition metal ferromagnets^38,39^.

A comparison of our ternary magnetic phase diagram with previous results is more challenging, because, as far as we know, no fully quantitative measurements of the saturation magnetization in Ni–Fe–Co ternary alloys have been performed so far. Qualitatively, however, our measurements are in good agreement with the saturation magnetization data obtained in electrodeposited films^40,41^, with the results from Ref. 40 allowing the most direct comparison.

## Methods

### Diffusion multiple sample fabrication

**Figure 7:**
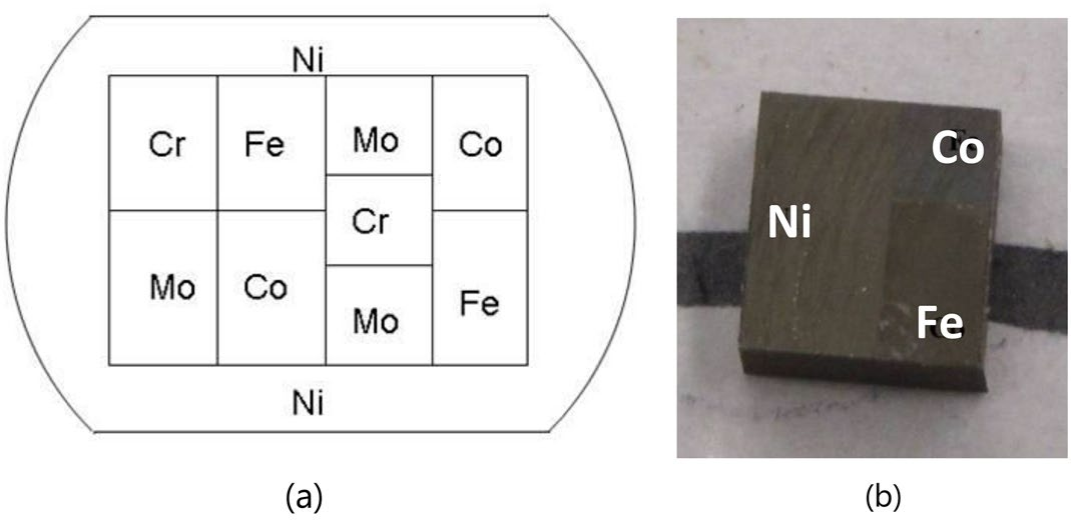
(**a**) The arrangement of the metal blocks in the diffusion multiple sample and (**b**) the micrograph of the Co–Fe–Ni diffusion triple after cutting. The contrast between the three areas is clearly seen.

Diffusion multiple samples were made by combining several blocks of pure iron, nickel and cobalt together and heating at 1000 °C for 1000 h to allow interdiffusion to form solid solutions in the diffusion zone. Initially, the pure metal pieces were cut into the required shapes and assembled together, and then the e-beam was welded into the final assembly. After hot isostatic pressing (HIP) treatment at 1000 °C and 200 MPa for 4 h (to ensure that the metal bars are in good thermal contact with each other), the sample was annealed at 1000 °C for 1000 h to create diffusion profiles of ~ 200 µm. After the heat treatment, the sample was quenched in water to retain the high-temperature phases. Wire EDM (electric discharge machining) was used to cut the sample into pieces that contained diffusion couple and multiple regions. We used part of the sample with the Fe–Co–Ni ternary region for the magnetic property analysis of one ternary and three binary systems (Fig. 7). The sample surface must be flat and free of mechanical deformations for effective EBSD analysis. Additionally, the top and bottom surfaces must be parallel to each other to ensure proper sample mounting in the SHPM. To remove the deformed surface layer originating from cutting, the samples were fine grinded and polished with SiC-Paper up to 1000#. Finally, oxide polishing with 0.05 µm colloidal silica was carried out to produce a nearly deformation-free surface. The final sample dimensions were approximately 4 mm × 3 mm × 2.5 mm, as shown in Fig. 7.

### SHPM measurements

The SHPM was set up in the *Quantum Design* Physical Property Measurements System (PPMS, Model 6000). Magnetization measurements were performed at room temperature in an applied external magnetic field of approximately 5 T along the *z*-direction. This field was sufficient to saturate the magnetization of all compositions of Co–Fe–Ni diffusion multiple samples. The Hall probe current (*I*_*H*_) was set at 100 μA. The Hall coefficient was approximately 5^.^10^–4^ Ω/Gauss with an electronics gain of 10^3^. While the signal-to-noise ratio (SNR) increased with the increase of the Hall current from 100 µA to 500 µA by about a factor of 2.5, the Hall voltage saturation (at 10 V) often occurred at 500 µA due to high values of applied magnetic fields and thus, all the measurements were conducted at *I*_*H*_ = 100 µA. The magnetic field sensitivity of the microscope (not the Hall probe itself) depended on the sample-probe separation. The spatial resolution of the SHPM measurements depended on the size of the Hall bar active area (0.8 µm × 0.8 µm), as well as the sample-probe distance, which was set to 10 μm. This relatively large distance was chosen to enable faster scan capabilities and to prevent the Hall probes from crushing during scans. Thus, the overall spatial resolution was approximately 10 μm, as determined primarily by the sample-probe distance. Additional discussion of the choice of measurement parameters and possible trade-offs is given in Supplemental Materials (Sect. 2).

## Conclusion

In this article, Scanning Hall probe microscopy was applied to investigate the composition-structure-magnetic properties of diffusion binary and ternary Co–Fe–Ni alloys fabricated by the diffusion multiple technique. We performed a comprehensive study of the crystalline structure of bulk Co–Fe–Ni magnetic alloys-using a combination of X-ray spectroscopy and electron back scatter diffraction—and the saturation magnetization of these alloys using pixel-by pixel characterization of a single sample representing a complete combinatorial materials library of these alloys with identical thermal history. The entire ternary magnetic phase diagram of the technologically important Co–Fe–Ni materials system over the full range of alloy compositions was mapped in a single experiment. The measured saturation magnetization dependence for the binary alloys was found to be consistent with the Slater-Pauling behavior. By using a significantly denser data point distribution than previously available, the maximum of the Slater-Pauling curve for the Co–Fe alloys was identified at ~ 32 at% of Co, consistent with ab initio calculations.

This approach is particularly useful for the saturation magnetization measurements, as, in contrast to the scanning SQUID magnetometer—which has an excellent magnetic field sensitivity but is limited to small fields—the SHPM technique can be applied in a large (several Tesla) external magnetic field required for saturation^17^. One can also envision performing temperature dependent measurement for some systems with lower Curie temperatures to identify composition dependent magnetic phase transitions. While the SHPM spatial resolution can be further improved by using the STM positioning technique, allowing one to bring the probe into a direct contact with the sample surface, this approach requires considerably more time and specialized techniques for sample preparation and measurements. Because of the inherent constraints of our approach in determining rapid changes in saturation magnetization within a narrow composition range, such as in the case of Invar^42^, for example (due to the influence of adjacent magnetic layers), it is not obvious that using the STM positioning technique is always beneficial. The comparative advantages and limitations of the SHPM technique are discussed in detail in the Supplemental section.

In summary, we have demonstrated that scanning Hall probe microscopy in concert with a combinatorial approach is a powerful high-throughput tool for the rapid characterization of magnetic materials, providing an effective metrology platform to optimize advanced magnetic alloys and to facilitate the search for new materials.

## Supplementary Information

Supplementary Information.

## References

[1] Xiang X-D, Sun X, Briceno G, Lou Y, Wang K-A, Chang H (1995). A combinatorial approach to materials discovery. Science.

[2] Wang J, Yao YK, Gao C, Takeuchi I, Sun X, Xiang X-D (1998). Identification of a blue photoluminescent composite material from a combinatorial library. Science.

[3] Sun X, Gao C, Wang J, Xiang X-D (1997). Identification and optimization of advanced phosphors using combinatorial libraries. Appl. Phys. Lett..

[4] Danielson E, Golden JH, McFarland EW, Reaves CM, Weinberg WH, Wu XD (1997). Combinatorial discovery of oxidative dehydrogenation catalysts within the Mo-V-Nb-O system. Nature.

[5] van Dover RB, Schneemeyer LF, Fleming RM (1998). Discovery of a useful thin-film dielectric using a composition-spread approach. Nature.

[6] Danielson E, Devenney M, Giaquinta DM, Golden JH, Haushalter RC, Mcfarland EW (1998). A rare-earth phosphor containing one-dimensional chains identified through combinatorial methods. Science.

[7] Sun X, Xiang X-D (1998). New phosphor (Gd_2−__x_Zn_x_)O_3−δ_:Eu_3+_ with high luminescent efficiency and superior chromaticity. Appl. Phys. Lett..

[8] Chang H, Gao C, Takeuchi I, Yoo Y, Wang J, Schultz PG (1998). Combinatorial synthesis and high throughput evaluation of ferroelectric/dielectric thin-film libraries for microwave applications. Appl. Phys. Lett..

[9] Chang H, Tacheuchi I, Xiang X-D (1999). A low-loss composition region identified from a thin-film composition spread of (Ba_1−x−__y_Sr_x_Ca_y_)TiO_3_. Appl. Phys. Lett..

[10] Xiang X-D (1999). Combinatorial materials synthesis and screening: an integrated materials chip approach to discovery and optimization of functional materials. Annu. Rev. Mater. Sci..

[11] Kuykendall T, Ulrich P, Aloni S, Yang P (2007). Complete composition tunability of InGaN nanowires using a combinatorial approach. Nat. Mater..

[12] Danielson E, Golden JH, McFarland EW, Reaves CM, Weinberg WH, Wu XD (1997). A combinatorial approach to the discovery and optimization of luminescent materials. Nature.

[13] Mallinson JC (1993). The Foundations of Magnetic Recording.

[14] Wysocki AL, Nguyen MC, Wang CZ, Ho KM, Postnikov AV, Antropov VP (2019). Concentration-tuned tetragonal strain in alloys: Application to magnetic anisotropy of FeNi_1−x_Co_x_. Phys Rev B.

[15] Slater JC (1936). The ferromagnetism of nickel II. Temperature effects. Phys. Rev..

[16] Pauling L (1938). The nature of the interatomic forces in metals. Phys. Rev..

[17] Yoo YK (2001). Continuous mapping of structure–property relations in Fe_1−x_Ni_x_ metallic alloys fabricated by combinatorial synthesis. Intermetallics.

[18] Yoo YK (2006). Identification of amorphous phases in the Fe–Ni–Co ternary alloy system using continuous phase diagram material chips. Intermetallics.

[19] Dolan GJ, Holtzberg F, Field C, Dinger TR (1989). Anisotropic vortex structure in Y_1_Ba_2_Cu_3_O_7_. Phys. Rev. Lett..

[20] Alers PB (1957). Structure of the intermediate state in superconducting lead. Phys. Rev..

[21] Murakami Y, Shindo D, Oikawa K, Kainuma R, Ishida K (2002). Magnetic domain structures in Co–Ni–Al shape memory alloys studied by Lorentz microscopy and electron holography. Acta Mater..

[22] Martin Y, Sugar D, Wickramasinghe HK (1988). High-resolution magnetic imaging of domains in TbFe by force microscopy. Appl. Phys. Lett..

[23] Goren RN, Tinkham M (1971). Patterns of magnetic flux penetration in superconducting films. J. Low Temp. Phys..

[24] Hilhorst A, Jacques PJ (2021). Diffusion multiples as a tool to efficiently explore the composition space of high entropy alloy. J. Phase Equilibr. Diffus..

[25] Zhao J-C, Zhao J-C (2007). Phase diagram determination using diffusion multiples. Methods for Phase Diagram Determination.

[26] Zhao J-C, Jackson MR, Peluso LA, Brewer LN (2002). A diffusion multiple approach for the accelerated design of structural materials. MRS Bull..

[27] Wei C, Zheng X, Cahill DG, Zhao J-C (2013). Micron resolution spatially resolved measurement of heat capacity using dual-frequency time-domain thermoreflectance. Rev. Sci. Instr..

[28] Zhao J-C, Zheng X, Cahill DG (2005). High-throughput diffusion multiples. Mater. Today.

[29] Ohnuma I (2002). Phase equilibria in the Fe–Co binary system. Acta Mater..

[30] Xiang X-D, Wang G, Zhang X, Xiang Y, Wang H (2015). Individualized pixel synthesis and characterization of combinatorial materials chip. Engineering.

[31] Kwiecien I, Wierzbicka-Mierni A, Szczerba M, Bobrowski P, Szulc Z, Wojewoda-Budka J (2021). On the disintegration of A1050/Ni201 explosively welded clads induced by long-term annealing. Materials.

[32] Aronova, M. *Combinatorial Investigation of Magnetic Materials*, Ph.D. Thesis, University of Maryland (2004).

[33] Bozorth RM (2003). Ferromagnetism.

[34] Bardos DI (1969). Mean magnetic moments in bcc Fe–Co alloys. J. Appl. Phys..

[35] Weiss P, Forrer RL (1929). saturation absolue des ferromagnétiques et les lois d'approche en fonction du champ et de la temperature. Ann. Phys..

[36] Schoen MAW, Lucassen J, Nembach HT, Silva TJ, Koopmans B, Back CH, Shaw JM (2017). Magnetic properties of ultrathin 3d transition-metal binary alloys. I. Spin and orbital moments, anisotropy, and confirmation of Slater-Pauling behavior. Phys. Rev. B.

[37] Schlosser WF (1973). Calculations of atomic volumes of Fe–Ni and Fe–Co alloys. Phys. Stat. Sol..

[38] Schwarz K, Mohn P, Blaha P, Kiibler J (1984). Electronic and magnetic structure of BCC Fe–Co alloys from band theory. J. Phys. F: Met. Phys..

[39] MacLaren JM, Schuthles TC, Butler WH, Sutton R, McHenry M (1999). Electronic structure, exchange interaction and Curie temperature of FeCo. J. Appl. Phys..

[40] Osaka T, Takai M, Hayashi K, Ohashi K, Saito M, Yamada K (1998). A soft magnetic CoNiFe film with saturation magnetic flux density and low coercivity. Nature.

[41] Yanai T, Shiraishi K, Akiyoshi T, Azuma K, Watanabe Y, Ohgai T, Morimura T, Nakano M, Fukunaga H (2016). Electroplated Fe–Co–Ni films prepared from deep-eutectic-solvent-based plating baths. AIP Adv..

[42] Chikazumi S, Mizoguchi T, Yamaguchi N (1968). The invar problem. J. Appl. Phys..

