# Supplementary material for: Magnetization–structure–composition phase diagram mapping in Co-Fe-Ni alloys using diffusion multiples and scanning Hall probe microscopy
Source: Sci Rep. 2022 Feb 4;12:1957. doi: 10.1038/s41598-022-05121-1 (PMC8816915; doi:10.1038/s41598-022-05121-1)
Supplement: Supplementary file 1 — Supplementary Information. [file 41598_2022_5121_MOESM1_ESM.docx]

**SUPLEMENTARY INFORMATION:**

1. **Hall Probe Calibration: Hall Probe Coefficients and Noise Level.**

It is important to calibrate the Hall probe before and after the measurements for the quantitative analysis, as some of the measurements take many hours and the values can drift. Calibrations are performed using the magnet in the PPMS system. The PPMS magnetic field accuracy is 0.02 mT, or 0.2 Gauss, which makes it suitable to use for Hall probe calibrations whose noise level at room temperature is around 10 Gauss. The Hall probe should be far away from the sample surface for proper calibration. We then ran a sequence of rectangular field pulses for about 30 seconds while performing a 1μm by 1μm Hall probe scan (away from the sample). Then, by using the SPM software, it is possible to extract the measured value of the field as well as the signal-to-noise ratio. The PPMS magnetic field was switching up and down between 0 and 100 Oe at the rate of ~ 0.1 Hz. The cross-sections along the white lines and the histograms reveal the measured magnetic field values and the noise level. The calibration results are presented in **Figure. S.1** at different temperatures, 300K in (Fig. S.2a) and 10K in (Fig. S.2b). From these measurements, the noise level was estimated as ~ 10 Gauss at 300K and ~2 Gauss at 10K.

(a)

(b)

Figure S.1. The results of the Hall probe calibration procedure at T = 300K (a); and T=10K (b) as described in the text.

1. **SHPM lateral resolution in the STM tracking and Lift-off modes**

Generally speaking, the SHPM *lateral resolution* is maximized by decreasing the size of the Hall Probe and the distance between the probe active region and the surface of the stray field sample. In addition to these two parameters, the lateral resolution is also dependent on the scan step size, which can be varied continuously from approximately 0.1 µm to 10 µm.

The LT-SHPM/STM from Nanomagnetics, Inc. can be used in two primary modes: the STM tracking mode, when the tunneling current feedback keeps the probe at a fixed nanometer-scale distance from the sample surface, and the lift – off mode, in which the Hall probe is fixed at the same (typically micron-scale) height above the sample surface. A typical LT-SHPM/STM scan obtained in the STM tracking mode is presented in Figure S.2, which shows images of domain structure in garnet thin film at three different temperatures. While the noise level decreases at lower temperature, the scan areas also decreases from 16 x 16 µm^2^ at 300K and 77K to ~ 7x7 µm^2^ at 5K.

Figure S.2. LT-SHPM scans of garnet thin film at different temperatures (cross sections along the lines and 3D views).

**Hall cross**


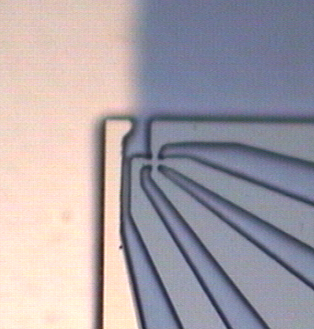


**STM Tip**

1. (b)

Figure S.3. (a) The SHPM Hall sensor assembly, with the GaAs/AlGaAs Hall probe; (b) The micrograph of the Hall probe with Hall cross (0.8 x 0.8 µm^2^) positioned at a distance of ~ 10 µm from the STM tip (the corner of the mesa). Images courtesy of *Nanomagnetics, Inc*.

The resolution of the Hall probe is primarily determined by size of the Hall bar (~0.8 x 0.8 µm^2^) and the distance of the 2D gas layer from the sample surface. In our case, due to the specifics of the Hall probe design and nanofabrication limitations, the Hall bar is about 10 µm from the corner of the Hall probe, which is used as an STM tip (see Fig. S.3b). Since the Hall probe is tilted by ~ 1.5^O^ with respect to the sample surface during scans, this distance translates into ~ 0.3 µm space separation between the Hall probe active area and the sample surface. Hence, the lateral resolution in the STM tracking mode is expected to be ~ 1 µm.

In the lift-off mode, used in our experiments, the lateral resolution is inferior to that of the STM tracking mode, as it is mostly determined by the Hall probe – sample surface separation, which was ~10 µm in our case. Hence, from the spatial resolution perspective alone, the STM tracking mode should have been employed. However, there are limitations that do not always justify the use of the STM tracking mode. Most importantly, the surface smoothness requirements for this mode are significantly more stringent, as the tunneling current between the surface and the tip of the Hall probe must be maintained at all times during the scan. In our experiments, due to the mechanical strain near the diffusion active regions, there are voids, typical of the diffusion multiple technique (see Fig. 4c, for example). In order to scan the entire surface with the STM tracking, the voids must be eliminated. This can be done by filling them up with a non-magnetic material, for example, followed by extensive high quality surface polishing and Au deposition. This procedure is not prohibitive, but very labor - intensive. In addition, while this would improve the resolution for most of the sample area, the SHPM images near the voids would still be distorted. Due to these reasons we have not used the STM tracking mode in our experiments on iffusion multiples. On the other hand, for materials combinatorial libraries prepared by different fabrication techniques, e.g. thin film deposition, the STM tracking mode may be preferable.

For a set Hall probe - sample separation, the lateral resolution of the SHPM technique is also dependent on the scan step size. In practice, to make sure that we are not reducing the lateral resolution much further, it is sufficient to choose the step size to be about half of the probe - sample distance (~ 5 µm in our experiments). Thus, the inversion technique assumption – that the magnetization is uniform within a single Hall probe step – works better when the probe - sample distance is minimized, as the spatial resolution is then improves and the step size is decreased. In our case, a 5 µm step size corresponds to ~ 50 points across the sample, as determined by the total lengths of the diffusion region (varying from 210 µm to 340 µm). This implies that the change of the diffusion couple composition within one step corresponds to a variation from ~ 1.5 at% to ~ 2.1 at% (a reasonable approximation). This was sufficient to obtain superior resolution compared to other techniques, but still allowed us to perform the entire scan in a single experiment.

1. **Tabulated prior saturation magnetization results**

Our experimental values for the Co-Fe-Ni diffusion sample are in good agreement with the accepted saturation magnetization results for pure Fe, Co and Ni, as shown in **Table S.1.**

|  |  | fcc | bcc | hcp |
| --- | --- | --- | --- | --- |
| Co | β/µ_B_ | 1.74 [Ref. ^[[1]](#endnote-1)^] | 1.70 [Ref. ^[[2]](#endnote-2)^] | 1.72 [Ref. ^[[3]](#endnote-3)^] |
|  | T_C_ (K) | 1394 [Ref. ^[[4]](#endnote-4)^] | 1450 [Ref. ^[[5]](#endnote-5)^] | 1070 [Ref. ^[[6]](#endnote-6)^] |
| Fe | β/µ_B_ | 0.7 [Ref. ^[[7]](#endnote-7)^] | 2.22 [Ref. 3] | 0.1 [Ref. ^[[8]](#endnote-8)^] |
|  | T_C_, T_N_^a^ (K) | 67^a^ [Ref. ^[[9]](#endnote-9)^, ^[[10]](#endnote-10)^] | 1043 [Ref. 3] | 100^a^ [Ref. 8] |
| Ni | β/µ_B_ | 0.62 [Ref. 7, ^[[11]](#endnote-11)^] | 0.52 [Ref. ^[[12]](#endnote-12)^] |  |
|  | T_C_ (K) | 625 [Ref. 7, 11] | 456 [Ref. 12] |  |

**Table S.1.** Magnetic properties (T_C_, T_N_, and β) of elemental Co, Fe, and Ni. ^a^ Neel Temperature.

**REFERENCES:**

1. Besnus, M.J., Meyer, A.J.P., Berninger, R. Magnetic moments and electron transfer in hexagonal Co-Cu alloys. *Phys. Lett.* *A*, **28**, 516-517 (1969). [↑](#endnote-ref-1)
2. Díaz-Ortiz, A. *et.al.* Structure and magnetism in bcc-based iron-cobalt alloys. *Phys. Rev.* B **73,** 224208 (2006). [↑](#endnote-ref-2)
3. Bozorth, R.M. *Ferromagnetism*, p. 867, New York: D. van Nostrand, (1951). [↑](#endnote-ref-3)
4. Myers, H.P., Sucksmith, W. The spontaneous magnetization of cobalt. *Proc. Roy. Soc. A*, 207-427 (1951). [↑](#endnote-ref-4)
5. Inden, G., Meyer, W.O. Approximate determination of the curie temperatures of bcc Fe-Co alloys. *Zeitschrift für Met.* **66**, 725-727 (1975). [↑](#endnote-ref-5)
6. Weiss, P., Forrer, R. La saturation absolue des ferromagnétiques et les lois d'approche en fonction du champ et de la temperature. Ann. Phys. **12**, 279-372 (1929). [↑](#endnote-ref-6)
7. Ohno, H. J. Antiferromagnetism in hcp Iron-Ruthenium and hcp Iron-Osmium Alloys. *Phys. Soc. Japan* **31**, 92-101 (1971). [↑](#endnote-ref-7)
8. Abrahams, S.C., Guttman, L., Kasper, J.S. Neutron Diffraction Determination of Antiferromagnetism in Face-Centered Cubic (γ) Iron. *Phys. Rev.* **127**, 2052 (1962). [↑](#endnote-ref-8)
9. Gonser, U., Meechan, C.J., Muir AH, Wiedersich H.

   Determination of Néel Temperatures in fcc Iron *J. Appl. Phys.* **34**, 2373 (1963). [↑](#endnote-ref-9)
10. Johanson, G.J., McGirr, M.B., Determination of the Néel Temperature of Face-Centered-Cubic Iron. *Phys Rev B* **1**, 3208 (1970). [↑](#endnote-ref-10)
11. Xiong, W. *et al*. Magnetic phase diagram of the Fe–Ni system. *Acta Materialia* **59**, 521–530 (2011). [↑](#endnote-ref-11)
12. Tian, C.S., Qian, D., Wu, D., He, R.H., Wu, Y.Z., Tang, W.X. *et al*. Body-Centered-Cubic Ni and Its Magnetic Properties. *Phys. Rev. Lett*. **94** 137210 (2005). [↑](#endnote-ref-12)
